# A machine learning-based prediction of hospital mortality in mechanically ventilated ICU patients

**DOI:** 10.1371/journal.pone.0309383

**Published:** 2024-09-04

**Authors:** Hexin Li, Negin Ashrafi, Chris Kang, Guanlan Zhao, Yubing Chen, Maryam Pishgar

**Affiliations:** Department of Industrial and Systems Engineering, University of Southern California (USC), Los Angeles, CA, United States of America; Atlantic Technological University, IRELAND

## Abstract

**Background:**

Mechanical ventilation (MV) is vital for critically ill ICU patients but carries significant mortality risks. This study aims to develop a predictive model to estimate hospital mortality among MV patients, utilizing comprehensive health data to assist ICU physicians with early-stage alerts.

**Methods:**

We developed a Machine Learning (ML) framework to predict hospital mortality in ICU patients receiving MV. Using the MIMIC-III database, we identified 25,202 eligible patients through ICD-9 codes. We employed backward elimination and the Lasso method, selecting 32 features based on clinical insights and literature. Data preprocessing included eliminating columns with over 90% missing data and using mean imputation for the remaining missing values. To address class imbalance, we used the Synthetic Minority Over-sampling Technique (SMOTE). We evaluated several ML models, including CatBoost, XGBoost, Decision Tree, Random Forest, Support Vector Machine (SVM), K-Nearest Neighbors (KNN), and Logistic Regression, using a 70/30 train-test split. The CatBoost model was chosen for its superior performance in terms of accuracy, precision, recall, F1-score, AUROC metrics, and calibration plots.

**Results:**

The study involved a cohort of 25,202 patients on MV. The CatBoost model attained an AUROC of 0.862, an increase from an initial AUROC of 0.821, which was the best reported in the literature. It also demonstrated an accuracy of 0.789, an F1-score of 0.747, and better calibration, outperforming other models. These improvements are due to systematic feature selection and the robust gradient boosting architecture of CatBoost.

**Conclusion:**

The preprocessing methodology significantly reduced the number of relevant features, simplifying computational processes, and identified critical features previously overlooked. Integrating these features and tuning the parameters, our model demonstrated strong generalization to unseen data. This highlights the potential of ML as a crucial tool in ICUs, enhancing resource allocation and providing more personalized interventions for MV patients.

## Introduction

In the United States, over one million patients receive mechanical ventilation (MV) annually in Intensive Care Units (ICU), occupying 24–41% of ICU beds at any given time [1]. Although MV is frequently considered a lifesaving intervention, patients undergoing non-surgical procedures that require MV have a hospital mortality rate exceeding 35% [2]. This high mortality rate underscores the necessity of studying the relationship between MV and mortality to develop predictive models that can aid in early intervention.

Current predictive models for MV patient mortality often lack sufficient accuracy and practical applicability. Existing studies often rely on limited features or fail to address data imbalance, reducing model effectiveness. This study aims to develop an improved predictive model to estimate the mortality of MV patients using patient health data, including early-stage symptoms [3]. The model is intended to assist ICU physicians in early alerting by utilizing a comprehensive database containing easily obtainable and well-generalized health data for better accuracy and prediction [4]. Previous research has demonstrated the feasibility of using patient health data for predictive modeling in ICU settings, highlighting the importance of integrating such models into clinical practice [5, 6].

There are many factors to consider when assessing risk for mechanically ventilated patients, including predictors available on the first day, which can be used to predict hospital mortality [7]. Effective machine learning prediction requires careful feature selection, accounting for the severity of the disease and outcomes to understand early prediction factors and the impact of MV [8, 9]. Previous studies have shown that XGBoost performs well in predicting hospital mortality, aiding in understanding different clinical situations for early-stage alerting [10, 11]. However, different models may be suitable for various purposes beyond performance, considering clinical outcomes and contributing factors, which can influence mortality rates [12, 13].

Most studies have not utilized advanced machine learning algorithms to their full potential or effectively addressed class imbalance issues, leaving a significant gap in the literature. This study’s key advantages include: (1) employing a comprehensive feature selection process using backward elimination, the Lasso method, and expert clinical insights; (2) effectively addressing class imbalances using SMOTE; (3) leveraging advanced algorithms known for superior handling of categorical variables and computational efficiency; and (4) applying rigorous data preprocessing techniques, such as mean imputation and label encoding for categorical transformations.

From the present study, CatBoost performed better than other approaches. Clinical situations and feature selection are crucial in assessing both short- and long-term factors [14–16]. By implementing machine learning approaches, we can better understand how these methods, combined with current healthcare data methodologies, improve the evaluation of risk factors [17, 18].

We thoroughly examined the inclusion criteria for our feature selection process using the MIMIC-III database when assessing risk factors, followed by various data extraction techniques for data preprocessing. Additionally, we implemented several machine learning models, each yielding unique results and offering different perspectives on predicting the mortality rate for MV patients.

## Methodology

### Data description

The Medical Information Mart for Intensive Care III (MIMIC-III) is a publicly accessible database [4]. It contains de-identified health information from over 40,000 ICU admissions at the Beth Israel Deaconess Medical Center, covering the years 2001 to 2012 [19]. Developed by the MIT Lab for Computational Physiology, MIMIC-III includes a wide range of data categories, such as demographics, vital signs, laboratory test results, medications, and mortality outcomes. This comprehensive database supports extensive research in clinical informatics.

### Patient selection

**Fig 1:**
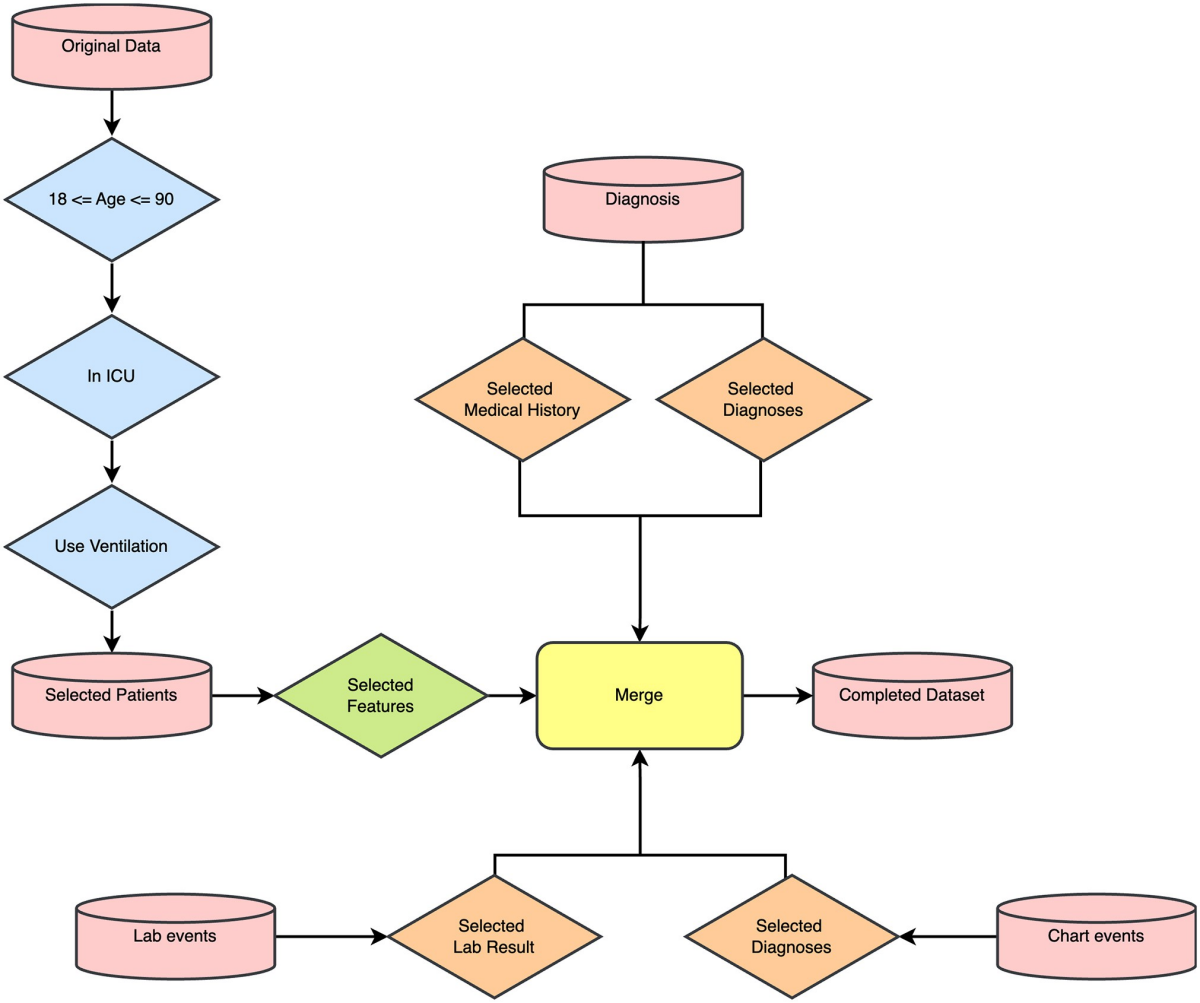
Patient selection. Flowchart illustrating the inclusion criteria and data preprocessing steps leading to the final cohort of 25,202 patient samples.

We initially included patients who required mechanical ventilation (MV) in the ICU. To exclude incomplete and duplicated data, we refined the dataset according to specific inclusion criteria: (I) patients aged between 18 and 90 years; (II) patients with complete mortality information; (III) patients with sufficient clinical data, ensuring columns with fewer than 90% missing data were included. We utilized ICD-9 codes to identify relevant patient records and linked these with ventilation-related data, resulting in a final cohort of 25,202 patient samples. The flowchart of patient selection and data preprocessing is illustrated in Fig 1.

### Feature selection and data preprocessing

The feature selection process in this study involved several stages. Initially, backward elimination and the Lasso method were employed to identify the most significant features [20, 21]. This selection was further refined through an extensive review of existing literature and clinical insights, resulting in the selection of 32 features.

The demographic data included age. Vital signs such as heart rate (HR), respiratory rate (RR), respiratory rate set (RR Set), temperature (TEMP), non-invasive blood pressure systolic (NIBP Systolic), non-invasive blood pressure diastolic (NIBP Diastolic), arterial blood pressure systolic (ABP Systolic), and arterial blood pressure diastolic (ABP Diastolic) were recorded. Additionally, laboratory values encompassing bicarbonate, serum creatinine, serum potassium concentration, and serum sodium concentration were included. Comorbidities such as liver failure, chronic heart failure, organ failure, sepsis, uncomplicated hypertension, and respiratory dysfunction were also documented. We included counts of specific diseases in our model based on literature and expert advice, which highlight their strong association with mortality in mechanically ventilated (MV) patients. This enhances our model’s performance by capturing the impact of these critical conditions. Differences in vital signs, such as the differences in heart rate, respiratory rate, NIBP systolic, and temperature, were calculated. The detailed overview of feature information used in this study is presented in Table 1.

**Table 1:** Detailed overview of feature information.

Feature Type	Feature Name	Feature Type	Feature Name
Lab Results	Bicarbonate_max_value	Vital Signs	Heart Rate_min
	Bicarbonate_min_value		Respiratory Rate_min
	Serum_Creatinine_min_value		Respiratory Rate Set_min
	Serum_Creatinine_diff		Temperature C (calc)_min
	Serum_Potassium_Concentration_max_value		Arterial BP [Diastolic]_min
	Serum_Sodium_Concentration_min_value		Heart Rate
	Serum_Sodium_Concentration_diff		Non Invasive Blood Pressure systolic_min
Demographics	Age		Non Invasive Blood Pressure diastolic_min
Comorbidities	liver_failure_count		Temperature Fahrenheit_min
	Chronic_heart_failure_count		Arterial BP [Systolic]_max
	organ_fail_count		NBP [Systolic]_max
	sepsis_count		Respiratory Rate Set_max
	Uncomplicated_hypertension_count		Arterial BP [Diastolic]_max
	respiratory_dysfunction_count		Temperature F_max
Differences	Heart Rate_diff		
	Respiratory Rate_diff		
	NBP [Systolic]_diff		
	Temperature F_diff		

To address class imbalances in the target variables, the Synthetic Minority Over-sampling Technique (SMOTE) was applied [22]. Data preprocessing involved removing columns with over 90% missing data and applying mean imputation to the remaining missing values. Categorical features were converted into numerical codes using a Label Encoder to integrate them into regression and machine learning models. These preprocessing steps were essential for standardizing the dataset, enabling efficient model training and evaluation, and ensuring that analyses accurately reflected the original measurements and categories present in the dataset.

### Model development and optimization

Our final dataset included 25,202 patients with 32 features. We performed a 70/30 train-test split to facilitate model evaluation, choosing this method over cross-validation to improve performance and computational efficiency. We developed and assessed several machine learning (ML) algorithms, including Logistic Regression, CatBoost, XGBoost, Decision Tree, Random Forest, Support Vector Machine (SVM), and K-Nearest Neighbors (KNN).

The performance of these models was primarily measured using the Area Under the Receiver Operating Characteristic (AUROC) scores, with accuracy and F1 scores also calculated for a comprehensive comparison. Given the widespread use of AUROC in existing literature, it was selected as the primary metric for model evaluation [23]. CatBoost emerged as the top performer, confirming its superior performance relative to other models. Introduced in 2017, CatBoost is a novel boosting method based on Gradient-Boosted Decision Trees (GBDT). It offers several advantages, including support for categorical variables, streamlined parameterization, fast prediction capabilities, and notable accuracy [24]. Our study highlights the advantages of using CatBoost in hospital mortality prediction.

To provide a thorough comparison, we included six widely employed machine learning algorithms as baseline models based on their frequent selection in the literature: Decision Tree, Random Forest, SVM, KNN, Logistic Regression, and XGBoost. This selection allowed us to evaluate the effectiveness of our data processing and feature selection methods comprehensively. We aimed to demonstrate that our overall approach could yield superior results across these models. Decision trees partition the feature space hierarchically, minimizing impurity in each split [25]. Random Forest, an ensemble method, aggregates multiple decision trees for prediction [26]. SVM seeks an optimal hyperplane for class separation, maximizing the margin [27]. KNN assigns class labels based on the majority vote among the k nearest neighbors [28]. Logistic Regression estimates binary outcome probabilities using a logistic function [29]. XGBoost, an accelerated gradient boosting implementation, iteratively enhances predictive accuracy. These diverse methodologies allowed us to comprehensively evaluate CatBoost’s performance [30].

In comparing CatBoost with these baseline models, distinct differences emerged [31]. CatBoost was included due to its distinct advantages, such as efficiently handling categorical features and mitigating overfitting through ordered boosting [32]. We hypothesized that CatBoost would generate a high-quality predictive model, ultimately enabling us to identify the best-performing model for our task. CatBoost’s symmetric algorithm for gradient-boosted decision trees provided significant enhancements in accuracy, robustness, and computational efficiency [33]. Evaluation metrics such as accuracy, precision, recall, F1-score, AUROC metrics, and calibration plots highlighted the predictive strengths of each model across various criteria. By employing these methodologies, we developed a robust predictive model for hospital mortality in mechanically ventilated ICU patients. This model offers valuable insights, supporting clinical decision-making and optimizing resource allocation within ICUs.

### Statistical analysis of models

To validate the statistical robustness of our model results, we conducted a comprehensive statistical analysis comparing the train and test sets using t-tests and chi-square tests. The dataset was split into a 70% train set and a 30% test set, which were used to evaluate the performance of trained models. A threshold p-value of 0.05 was set to determine the significance of differences [34]. This analysis focused on identifying any significant discrepancies between the two datasets, ensuring the reliability of the study’s findings.

T-tests were employed to compare the means of continuous variables between the train and test sets. For categorical variables, chi-square tests were utilized to assess the independence between the two sets. We tested the hypothesis that there was no significant difference between the train and test sets. If the p-value for each variable was greater than 0.05, we could reject the null hypothesis and conclude that the test set had notable distinctions from the train set. This analysis was crucial for confirming the consistency and generalizability of our model.

### Features importance

We analyzed each variable’s impact on our model using SHAP (SHapley Additive exPlanations) values, which measure feature importance. SHAP values are particularly useful in the medical domain as they provide clear and interpretable insights into model predictions, aiding healthcare professionals in understanding the reasoning behind diagnostic decisions [35, 36]. SHAP values reveal the most influential features in predicting the model’s output by ranking them based on their impact [37].

**Fig 2:**
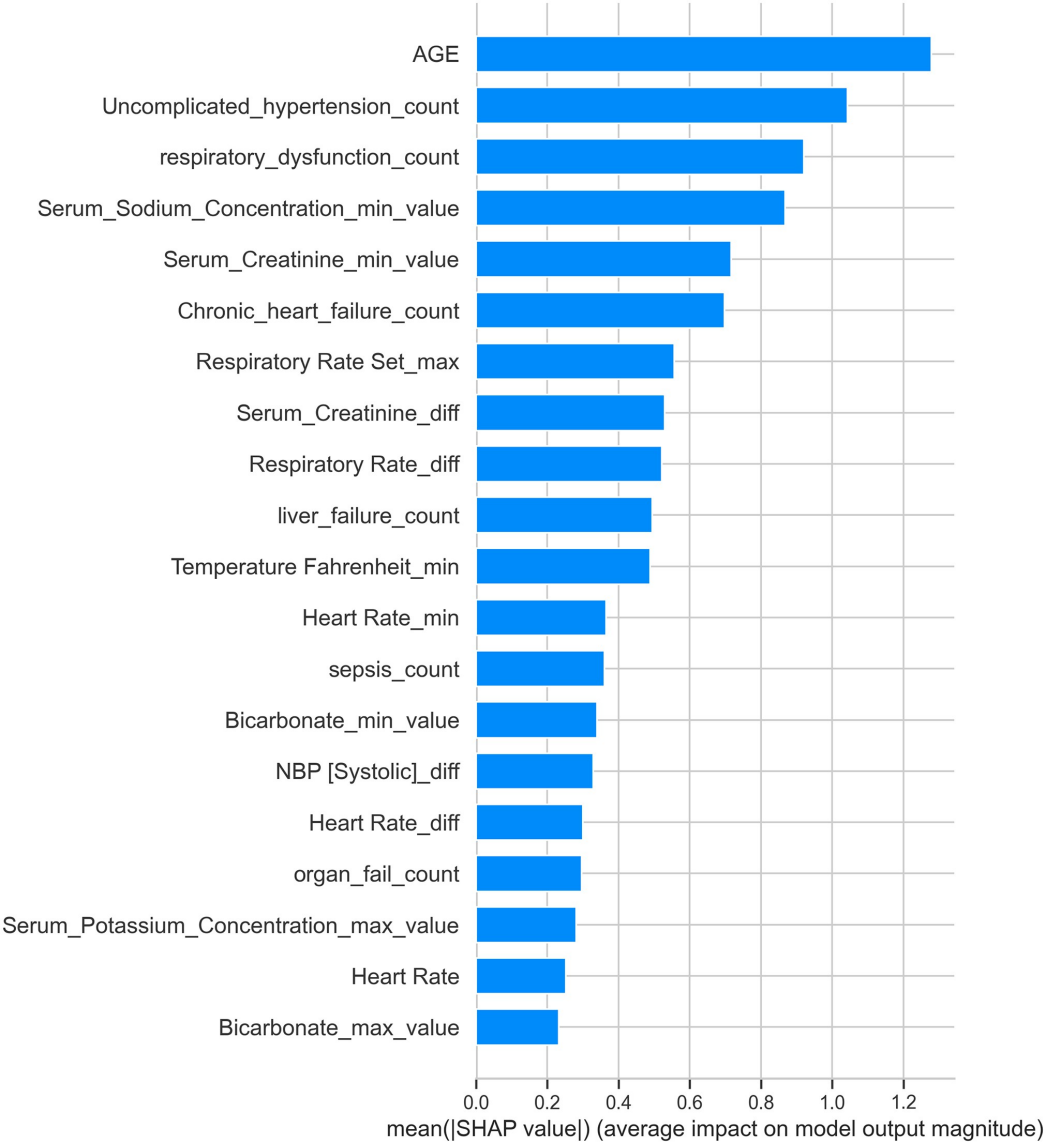
SHAP analysis. Bar plot of mean absolute SHAP values, demonstrating the impact of each feature on the model’s predictions, with ‘Age’ being the most significant, followed by ‘Uncomplicated_hypertension_count’ and ‘respiratory_dysfunction_count’.

The graphical representation of SHAP values includes a bar plot of mean absolute SHAP values, indicating how much each feature contributes to the model’s predictions. For instance, ‘Age’ was identified as the most significant feature, followed by ‘Uncomplicated_hypertension_count’ and ‘respiratory_dysfunction_count’. These top-ranked features had higher mean absolute SHAP values, showing their significant impact on the model’s output. Lesser-impact features, such as ‘Heart_Rate’ and ‘Bicarbonate_max_value’, had lower SHAP values. The SHAP summary plot, presented in Fig 2, visually demonstrates these findings. This analysis provides valuable insights into the internal mechanics of our machine learning models, ensuring transparency, improving model accuracy, and informing decision-making by highlighting key drivers of predictions.

This graphical representation provides a valuable tool for understanding the inner workings of complex ML models. By interpreting the average impact of each feature on model output magnitude, we gain insights into which features drive predictions the most and help ensure transparency in the predictive process. This, in turn, aids in debugging, improving model accuracy, and making informed decisions. This analysis provides valuable insights into the internal mechanics of our machine learning models, ensuring transparency, improving model accuracy, and informing decision-making by highlighting key drivers of predictions.

## Results

### Cohort characteristics model completion

Following our approach for feature selection and data preprocessing for ICU patients requiring mechanical ventilation, our final dataset included 25,202 patients from the MIMIC-III database. The selected cohort was randomly split into training and testing sets with a ratio of 70/30, resulting in 17,641 patients for training and 7,561 patients for test. The training set was used to train the models, while the testing set was employed to evaluate the performance of our proposed model. To rigorously evaluate the disparity between the training and testing sets, we conducted a statistical analysis focusing on the hypothesis that the testing set exhibited notable distinctions from the training set. A threshold p-value of 0.05 was set to ascertain the significance of the differences. The analysis aimed to identify any substantial gaps between the two datasets, ensuring the reliability of the study’s findings. The detailed cohort values and p-values reflecting differences between the training and testing sets are presented in Table 2.

**Table 2:** Detailed overview of cohort characteristics for train and test cohort. Values are presented as means with the standard deviations in parentheses.

Characteristics	Train Cohort (N = 17,638)	Test Cohort (N = 7,562)	T-Stat	P-Value
Age	63.12(16.50)	62.97(16.41)	0.68	0.49
Respiratory Rate_min	8.31(4.23)	8.35(4.21)	-0.6	0.55
Respiratory Rate Set_min	10.12(3.72)	10.07(3.76)	1.18	0.24
Temperature C (calc)_min	35.53(2.38)	35.53(2.4)	0.21	0.83
Arterial BP [Diastolic]_min	33.46(13.95)	33.49(14.18)	-0.15	0.88
Non Invasive Blood Pressure systolic_min	86.08(11.99)	86.12(11.65)	-0.22	0.82
Non Invasive Blood Pressure diastolic_min	40.42(8.05)	40.33(7.97)	0.93	0.35
Temperature Fahrenheit_min	94.02(8.23)	93.98(8.43)	1.13	0.26
Heart Rate_min	60.02(16.77)	60.07(16.79)	-0.2	0.84
Arterial BP [Systolic]_max	166.22(22.91)	165.72(23.44)	1.58	0.11
NBP [Systolic]_max	150.96(22.11)	150.57(22.33)	1.31	0.19
Respiratory Rate Set_max	16.46(4.72)	16.42(4.71)	0.71	0.48
Temperature F_max	100.32(1.95)	100.36(2.07)	0.71	0.48
Arterial BP [Diastolic]_max	92.73(20.80)	92.39(20.59)	1.12	0.23
Heart Rate	102.97(6.02)	101.98(14.46)	1.14	0.26
NBP [Systolic]_diff	69.82(36.85)	69.79(36.73)	0.06	0.95
Respiratory Rate_diff	25.36(11.35)	25.16(11.34)	1.29	0.2
Temperature F_diff	5.52(9.69)	5.42(9.17)	0.81	0.42
Heart Rate_diff	58.12(27.83)	58.02(27.67)	0.28	0.78
Bicarbonate_max_value	30.32(4.64)	30.36(4.92)	-0.53	0.6
Bicarbonate_min_value	20.17(4.59)	20.19(4.61)	-0.38	0.7
Serum_Creatinine_min_value	0.80(0.67)	0.80(0.71)	0.37	0.71
Serum_Creatinine_diff	69.63(312.66)	66.18(336.43)	0.78	0.43
Serum_Potassium_Concentration_max_value	12.13(17.83)	11.83(17.04)	1.23	0.22
Serum_Sodium_Concentration_min_value	106.60(45.45)	106.59(45.36)	0.03	0.98
Serum_Sodium_Concentration_diff	38.07(47.77)	38.21(49.47)	-0.19	0.85
liver_failure_count	0.07(0.33)	0.07(0.32)	0.5	0.62
Chronic_heart_failure_count	0.14(0.56)	0.12(0.5)	2.17	0.03
organ_fail_count	1.39(2.12)	1.35(1.88)	1.58	0.11
sepsis_count	0.15(0.44)	0.15(0.43)	0.56	0.58
Uncomplicated_hypertension_count	0.75(0.70)	0.76(0.74)	-0.67	0.5
Respiratory dysfunction_count	0.30(0.62)	0.30(0.6)	0.45	0.65

The results indicated that the training and testing sets were largely comparable. However, one variable, ‘Chronic_heart_failure_count,’ reflected a significant difference between the datasets, as evidenced by its p-value. Overall, the analysis confirmed that, apart from this variable, there were no significant differences between the training and testing sets, ensuring that the model’s results are reliable and generalizable.

### Evaluation metrics proposed and baseline models’ performance

**Fig 3:**
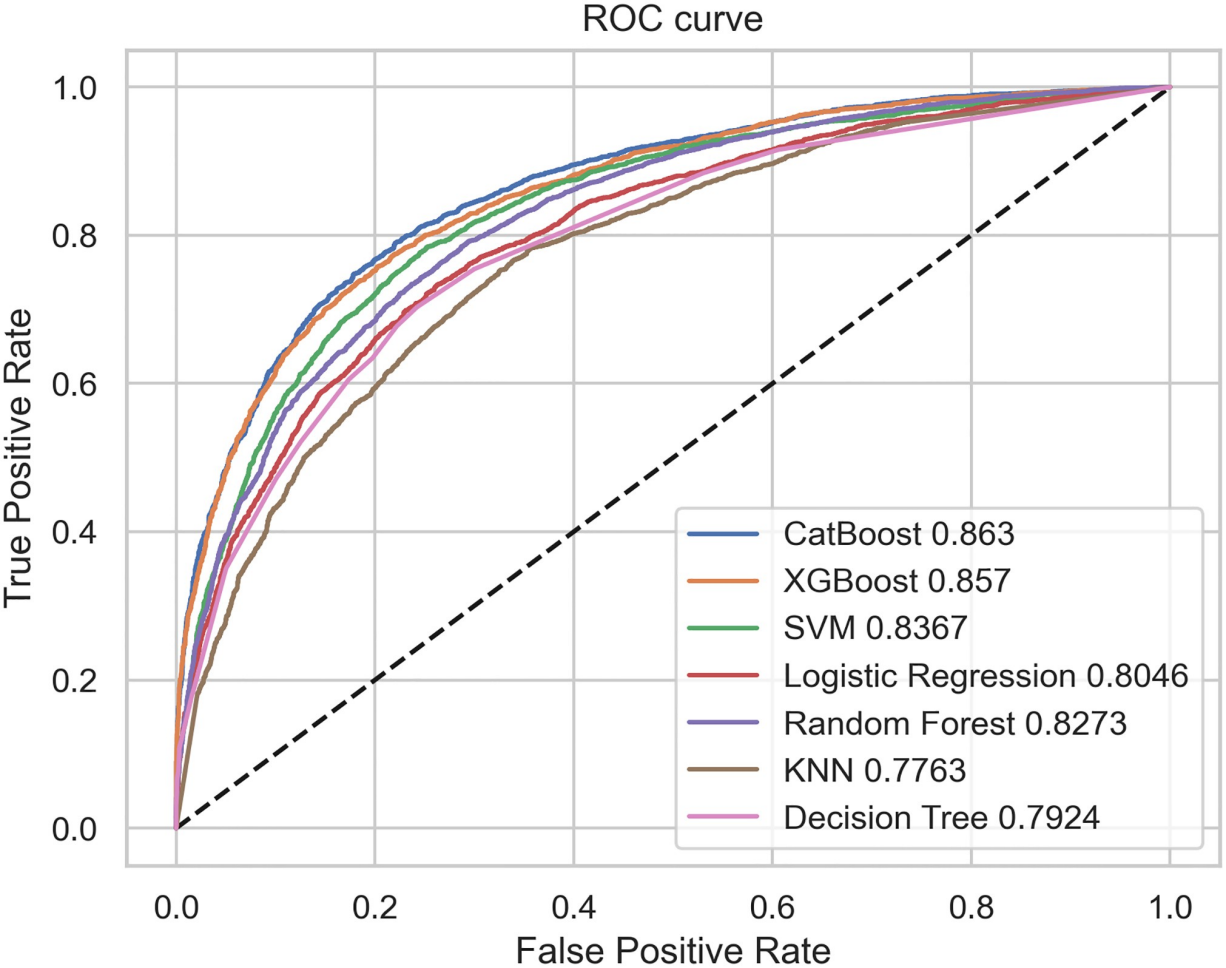
Model comparison. The AUROC curves and scores for seven models: CatBoost, XGBoost, SVM, Logistic Regression, Random Forest, KNN, and Decision Tree.

The results for both the proposed and baseline models are summarized in Table 3. The proposed approach, using the CatBoost model, achieved an AUROC of 0.862, an accuracy score of 0.789, and an F1 score of 0.747. These metrics highlight the model’s accuracy and robustness in reducing both Type I and Type II errors, validating its effectiveness for our predictive modeling tasks. The plot of the ROC curves for both the proposed and baseline models is presented in Fig 3.

**Table 3:** Summary of the evaluation metrics (AUCROC, Accuracy, Precision, Recall, F-Score) for the prediction models on the test set.

Model	AUROC	Accuracy	Precision	Recall	F-Score
Decision Tree	0.793	0.734	0.697	0.678	0.687
Random Forest	0.826	0.750	0.708	0.714	0.711
KNN	0.776	0.714	0.624	0.654	0.663
Logistic Regression	0.805	0.737	0.689	0.706	0.697
SVM	0.837	0.766	0.721	0.745	0.732
XGBoost	0.857	0.784	0.766	0.717	0.741
CatBoost (Proposed)	0.862	0.789	0.771	0.724	0.747

Among the baseline models developed using the MIMIC-III database, the Support Vector Machine (SVM) and XGBoost models performed notably well, with high AUROC, accuracy, precision, recall, and F1 scores. In contrast, the Decision Tree and K-Nearest Neighbors (KNN) models showed relatively lower performance across these metrics. Overall, the CatBoost model emerged as the top-performing model, followed closely by SVM and XGBoost. The results indicate that the proposed approach outperforms the best baseline models.

**Fig 4:**
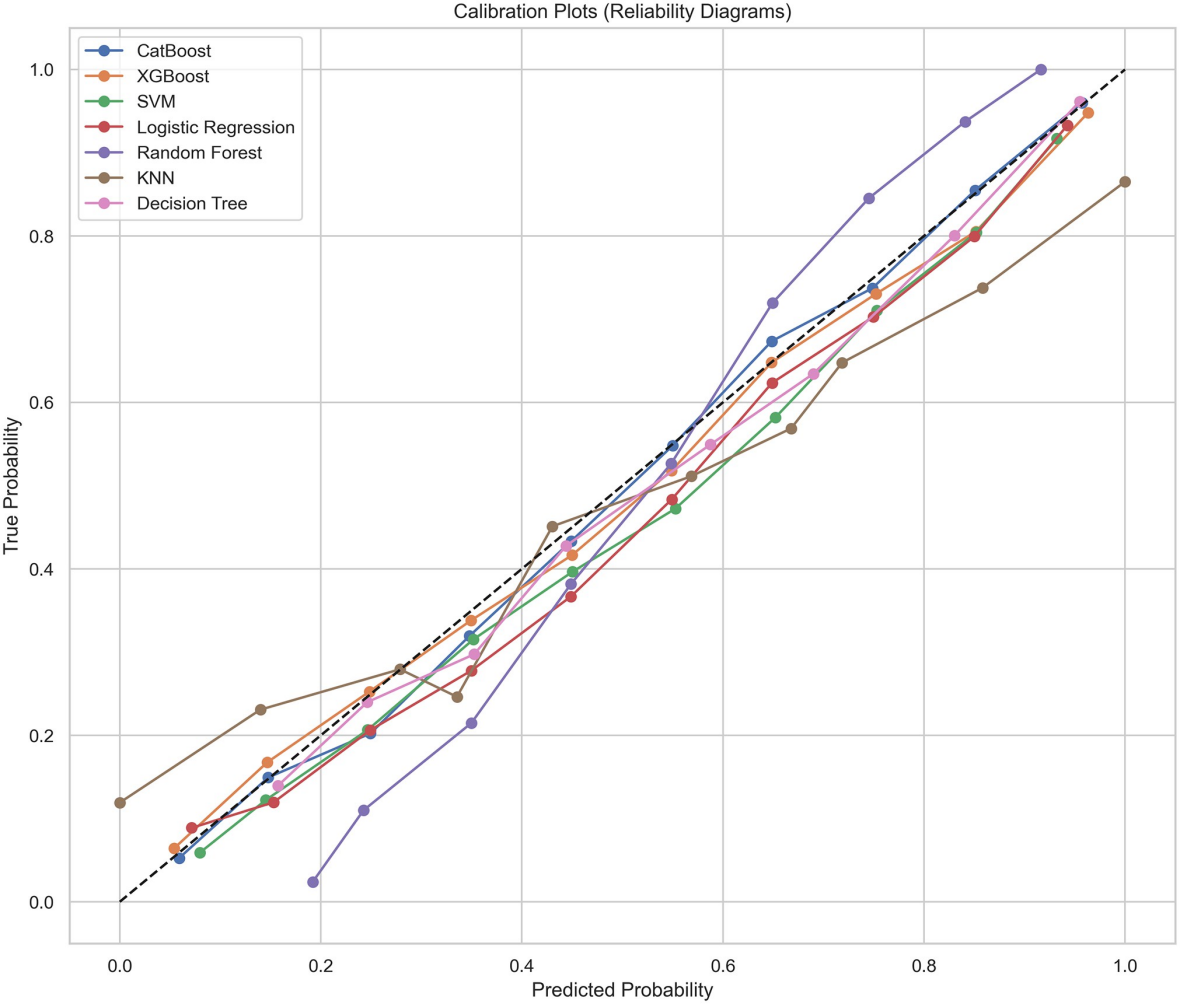
Calibration curves. We used calibration techniques for seven machine learning models, comparing predicted probabilities with true probabilities. CatBoost, XGBoost, and Logistic Regression show better calibration, while KNN and Decision Tree show poorer calibration.

Fig 4 shows the calibration curves for seven machine learning models: CatBoost, XGBoost, SVM, Logistic Regression, Random Forest, KNN, and Decision Tree. The X-axis represents the predicted probability, and the Y-axis represents the true probability, with the diagonal line indicating perfect calibration. Observations reveal that CatBoost, XGBoost, and Logistic Regression exhibit good calibration, closely aligning with the diagonal line. SVM and Random Forest show moderate calibration, with some deviation. In contrast, KNN and Decision Tree exhibit poor calibration, slightly deviating from the diagonal line. These results suggest that CatBoost, XGBoost, and Logistic Regression are better at predicting accurate probabilities.

## Discussion

### Summary of existing model compilation

Several models have been concurrently developed to predict mortality for ICU patients, specifically those receiving mechanical ventilation (MV). These include machine learning frameworks using various techniques and databases, with a particular focus on the MIMIC-III database due to its comprehensive nature.

Kim et al. demonstrated that the XGB model outperformed conventional scoring systems like APACHE II and ProVent in predicting 30-day mortality for mechanically ventilated patients, achieving an AUC of 0.79 [38]. Mamandipoor et al. showed that an RNN-based model had higher predictive performance for respiratory disorder patients in the ICU, with an AUC of 0.75 when evaluating patients admitted with respiratory disorders, based on 50 features [39]. Yu et al. developed a CatBoost model to predict in-hospital mortality for MV patients with COVID-19, achieving an AUC of 0.90. The model utilized demographic information, vital signs, and laboratory values from the ER [10]. George et al. created a deep learning model to predict 3-month mortality in patients requiring more than 7 days of mechanical ventilation, achieving an AUC of 0.74, which outperformed the ProVent model (AUC 0.59) under similar conditions, using 250 features [19].

Notably, Zhu et al. utilized a wide range of features and ML methods, ultimately finding that the XGBoost model performed best with the highest AUC among their tested models. In Zhu’s study, which also utilized the MIMIC-III database, data preprocessing resulted in a dataset of 25,659 patients and 55 predictors. Their features included demographics, ICU diagnosis, pre-ICU comorbidities, vital signs, disease severity scores, and laboratory test results from the first day of ICU admission. Various machine learning methods were employed, including KNN, bagging, logistic regression, decision tree, XGBoost, random forest, and neural networks [7].

In contrast to the existing literature, our study employs a more streamlined feature selection process, using only 32 features derived from backward elimination, the Lasso method, and expert clinical insights. We addressed class imbalances using the Synthetic Minority Over-sampling Technique (SMOTE) and managed missing data with median imputation and categorical transformations via Label Encoder. Our proposed approach leverages the CatBoost model, known for its superior handling of categorical variables and computational efficiency. The CatBoost model in our study achieved an AUROC of 0.862, a significant improvement from the initial 0.821 AUROC score, and demonstrated notable performance in terms of accuracy (0.789) and F1-score (0.747). This is a marked improvement over Zhu et al.’s findings, which, despite using more features (55 in total), did not achieve as high an AUROC score. Additionally, the calibration plots showed that our models are well-calibrated.

The key advantages of our approach over previous studies are multifold: (1) our systematic feature selection process, combined with clinical insights, ensures that only the most pertinent features are included, reducing complexity and enhancing model accuracy; (2) the application of SMOTE effectively addresses the imbalance in target variables, which was not considered in previous studies; and (3) CatBoost’s gradient boosting architecture and efficiency in handling high-dimensional data make it a superior choice for this application, outperforming traditional models in both accuracy and computational speed.

### Study limitations and future research

Our study has several limitations that need to be acknowledged. Firstly, we were unable to validate our model using external datasets due to the lack of access to comprehensive databases similar to MIMIC-III. This limitation restricts our ability to confirm the generalizability and robustness of our proposed model across different patient populations and clinical settings. Future research should focus on validating our models using external datasets to ensure their applicability in diverse healthcare environments. This includes testing the model’s performance metrics, such as AUROC, accuracy, and F1-score, on new data to confirm consistency and identifying any biases or limitations in different patient populations. Integrating real-time data streams and employing continuous learning models will ensure the model remains current with the latest clinical data and practices, enhancing its predictive accuracy and utility.

Secondly, the MIMIC-III database we utilized is over ten years old and lacks comprehensive historical information for many patients. This could introduce bias in our dataset selection, potentially affecting the model’s performance and its relevance to current clinical practices. The reliance on an older database may not fully capture the advancements in ICU care and patient management that have occurred over the past decade. To address this limitation, future research should aim to leverage newer databases that reflect the latest clinical data and practices, thereby improving the model’s accuracy and applicability.

Additionally, while our study implemented advanced feature selection and data preprocessing techniques, there is still room for improvement in handling missing data and imbalances in the dataset. Future studies could explore more sophisticated imputation methods and balancing techniques to further enhance model performance. By addressing these limitations, future research can build on our findings and contribute to more robust and generalizable predictive models for ICU patient outcomes.

To enhance the accuracy of the CatBoost predictive model, several strategies can be employed: (1) Grid search or Bayesian optimization can identify optimal parameters, improving performance, although this process can be computationally expensive and time-consuming. (2) Feature engineering, involving new features based on domain knowledge or techniques like feature interaction, can significantly boost accuracy, but it relies on domain expertise and may introduce bias if not done carefully. (3) Incorporating ensemble methods to combine predictions from multiple models can reduce overfitting and enhance predictive accuracy, yet it increases model complexity, making it harder to interpret and requiring more computational resources. Despite these limitations, these strategies collectively enhance the accuracy and robustness of the CatBoost predictive model.

Future research should explore the integration of deep learning approaches, such as Convolutional Neural Networks (CNNs) and Recurrent Neural Networks (RNNs), which can provide significant improvements in handling complex and high-dimensional data. In this study, we used maximum, average, and minimum values of laboratory data as indicators of patient conditions. However, this approach overlooks important time series information, such as data fluctuations and trends. Incorporating time series analysis could capture these temporal patterns, enhancing the model’s performance and generalizability. Time series methods can identify rapid changes in parameters often associated with high mortality rates, leading to more precise and reliable predictions.

Furthermore, it is essential to consider the clinical implementation of predictive models. Developing user-friendly tools and interfaces that can be seamlessly integrated into clinical workflows will be crucial. Engaging with clinicians during the development process to understand their needs and preferences will ensure that the models are not only accurate but also practical and easy to use in everyday clinical practice. Additionally, understanding privacy perceptions and behavior is crucial when dealing with sensitive health data, ensuring ethical standards and patient trust [40, 41]. This approach will ultimately enhance the adoption and impact of predictive models in improving patient outcomes in the ICU.

Lastly, future studies should investigate the potential of employing real-time data streams and continuous learning models to keep the predictive systems up-to-date with the latest clinical data and practices. This dynamic approach could significantly improve the model’s responsiveness and accuracy, ensuring that it remains relevant and effective in rapidly evolving clinical environments.

## Conclusion

This study significantly improves the prediction of hospital mortality for ICU patients on mechanical ventilation using advanced machine learning techniques. By applying data imputation, bootstrapping, and model optimization, we enhanced our models’ predictive accuracy and robustness, as shown by the substantial improvements in AUC and accuracy metrics.

The data preprocessing methodology significantly reduced the number of relevant features, simplifying computational processes, and identified critical features previously overlooked. Integrating these features and tuning the parameters, our model demonstrated strong generalization to unseen data.

The CatBoost model, noted for its efficient handling of categorical variables and computational demands, proved particularly effective. Our streamlined feature selection process and the use of SMOTE ensured the inclusion of relevant features, reducing complexity and boosting model performance. The CatBoost model achieved an AUROC of 0.862, an accuracy of 0.789, and an F1-score of 0.747, significantly outperforming other models tested. Our streamlined feature selection process, employing backward elimination, the Lasso method, and expert clinical insights, ensured the inclusion of 32 relevant features. The use of SMOTE addressed class imbalances effectively, further boosting model performance. Additionally, our models demonstrated good calibration, ensuring that predicted probabilities closely matched actual outcomes.

The key results of this study include enhanced predictive accuracy, effective feature handling, robust feature selection, and improved calibration. These findings highlight the potential of leveraging advanced machine learning techniques to aid clinicians in making more informed decisions, ultimately improving patient outcomes in ICUs. Continued research and validation with diverse datasets will further enhance the applicability and reliability of these predictive models across different clinical settings.

## Supporting information

S1 Graphical abstract (PDF)

## References

[1] Kempker JA, Abril MK, Chen Y, Kramer MR, Waller LA, Martin GS. The epidemiology of respiratory failure in the United States 2002–2017: a serial cross-sectional study. Crit Care Explor. 2020;2:e0128. doi: 10.1097/CCE.0000000000000128 32695994 PMC7314331

[2] Wunsch H, Wagner J, Herlim M, Chong DH, Kramer AA, Halpern SD. Occupancy and mechanical ventilator use in the United States. Crit Care Med. 2013;41:2712–2719. 23963122 10.1097/CCM.0b013e318298a139 PMC3840149

[3] Mehta AB, Syeda SN, Wiener RS, Walkey AJ. Epidemiological trends in invasive mechanical ventilation in the United States: A population-based study. J Crit Care. 2015;30(6):1250–1256. doi: 10.1016/j.jcrc.2015.07.007 26271686 PMC4628853

[4] Johnson A, Pollard T, Mark R. MIMIC-III Clinical Database (version 1.4). PhysioNet. 2016.

[5] Gao J, Lu Y, Ashrafi N, Domingo I, Alaei K, Pishgar M. Prediction of Sepsis Mortality in ICU Patients Using Machine Learning Methods. medRxiv. 2024;2024.03.14.24304184. doi: 10.1186/s12911-024-02630-z 39152423 PMC11328468

[6] Zhang J, Li H, Ashrafi N, Yu Z, Placencia G, Pishgar M. Prediction of In-Hospital Mortality for ICU Patients with Heart Failure. medRxiv. 2024;2024.06.25.24309448.

[7] Zhu Y, Zhang J, Wang G, Yao R, Ren C, Chen G, et al. Machine Learning Prediction Models for Mechanically Ventilated Patients: Analyses of the MIMIC-III Database. Front Med. 2021;8:662340. doi: 10.3389/fmed.2021.662340 34277655 PMC8280779

[8] Su L, Zhang Z, Zheng F, et al. Five novel clinical phenotypes for critically ill patients with mechanical ventilation in intensive care units: a retrospective and multi database study. Respir Res. 2020;21:325. doi: 10.1186/s12931-020-01588-6 33302940 PMC7727781

[9] Liang Y, Zhu C, Tian C, et al. Early prediction of ventilator-associated pneumonia in critical care patients: a machine learning model. BMC Pulm Med. 2022;22:250. doi: 10.1186/s12890-022-02031-w 35752818 PMC9233772

[10] Yu L, Halalau A, Dalal B, Abbas AE, Ivascu FA, Amin M, et al. Machine learning methods to predict mechanical ventilation and mortality in patients with COVID-19. PLoS One. 2021;16(4):e0249285. doi: 10.1371/journal.pone.0249285 33793600 PMC8016242

[11] Yao RQ, Jin X, Wang GW, Yu Y, Wu GS, Zhu YB, et al. A machine learning-based prediction of hospital mortality in patients with postoperative sepsis. Front Med (Lausanne). 2020;7:445. doi: 10.3389/fmed.2020.00445 32903618 PMC7438711

[12] Hsu PC, Lin YT, Kao KC, et al. Risk factors for prolonged mechanical ventilation in critically ill patients with influenza-related acute respiratory distress syndrome. Respir Res. 2024;25:9. doi: 10.1186/s12931-023-02648-3 38178147 PMC10765923

[13] Dai Z, et al. Analysis of adult disease characteristics and mortality on MIMIC-III. PLoS One. 2020;15(4):e0232176. doi: 10.1371/journal.pone.0232176 32353003 PMC7192440

[14] Lin Z, Huang X, Shan X. Development and validation of a survival prediction model for patients received mechanical ventilation in the intensive care unit: a large sample size cohort from the MIMIC database. Ann Palliat Med. 2022;11(6):2071–2084. doi: 10.21037/apm-22-646 35817742

[15] Sayed M, Riaño D, Villar J. Predicting Duration of Mechanical Ventilation in Acute Respiratory Distress Syndrome Using Supervised Machine Learning. J Clin Med. 2021;10(17):3824. doi: 10.3390/jcm10173824 34501270 PMC8432117

[16] Li L, Zhang Z, Xiong Y, Hu Z, Liu S, Tu B, et al. Prediction of hospital mortality in mechanically ventilated patients with congestive heart failure using machine learning approaches. Int J Cardiol. 2022; Version of Record 16 May 2022. doi: 10.1016/j.ijcard.2022.04.063 35483478

[17] Van Calster B, Wynants L, Timmerman D, Steyerberg EW, Collins GS. Predictive analytics in healthcare: how can we know it works? J Am Med Inform Assoc. 2019;26(12):1651–1654. doi: 10.1093/jamia/ocz130 31373357 PMC6857503

[18] Harutyunyan H, Khachatrian H, Kale DC, Ver Steeg G, Galstyan A. Multitask learning and benchmarking with clinical time series data. Sci Data. 2019;6:96. doi: 10.1038/s41597-019-0103-9 31209213 PMC6572845

[19] George N, Moseley E, Eber R, Siu J, Samuel M, Yam J, et al. Deep learning to predict long-term mortality in patients requiring 7 days of mechanical ventilation. PLoS One. 2021;16(6):e0253443. doi: 10.1371/journal.pone.0253443 34185798 PMC8241081

[20] Sauer CM, Sasson D, Paik KE, McCague N, Celi LA, Sánchez Fernández I, et al. Feature selection and prediction of treatment failure in tuberculosis. PLoS One. 2018;13(11):e0207491. doi: 10.1371/journal.pone.0207491 30458029 PMC6245785

[21] van Egmond MB, Spini G, van der Galien O, IJpma A, Veugen T, Kraaij W, et al. Privacy-preserving dataset combination and Lasso regression for healthcare predictions. BMC Med Inform Decis Mak. 2021;21(1):266. doi: 10.1186/s12911-021-01582-y 34530824 PMC8445286

[22] Elreedy D, Atiya AF. A comprehensive analysis of synthetic minority oversampling technique (SMOTE) for handling class imbalance. Inf Sci. 2019;505:32–64. doi: 10.1016/j.ins.2019.07.070

[23] Parodi S, Verda D, Bagnasco F, Muselli M. The clinical meaning of the area under a receiver operating characteristic curve for the evaluation of the performance of disease markers. Epidemiol Health. 2022;44. doi: 10.4178/epih.e2022088 36265519 PMC10089712

[24] Prokhorenkova L, Gusev G, Vorobev A, Dorogush AV, Gulin A. CatBoost: unbiased boosting with categorical features. Adv Neural Inf Process Syst. 2018;31:6638–6648.

[25] Seto H, Oyama A, Kitora S, et al. Gradient boosting decision tree becomes more reliable than logistic regression in predicting probability for diabetes with big data. Sci Rep. 2022;12:15889. doi: 10.1038/s41598-022-20149-z 36220875 PMC9553945

[26] Mbonyinshuti F, Nkurunziza J, Niyobuhungiro J, Kayitare E. Application of random forest model to predict the demand of essential medicines for non-communicable diseases management in public health facilities. Pan Afr Med J. 2022;42:89. doi: 10.11604/pamj.2022.42.89.33833 36034003 PMC9379432

[27] Zhou X, Li X, Zhang Z, et al. Support vector machine deep mining of electronic medical records to predict the prognosis of severe acute myocardial infarction. Front Physiol. 2022;13:991990. doi: 10.3389/fphys.2022.991990 36246101 PMC9558165

[28] Xing W, Bei Y. Medical Health Big Data Classification Based on KNN Classification Algorithm. IEEE Access. 2019.

[29] Schober P, Vetter TR. Logistic Regression in Medical Research. Anesth Analg. 2021;132(2):365–366. doi: 10.1213/ANE.0000000000005247 33449558 PMC7785709

[30] Zhang Z, Zhao Y, Canes A, Steinberg D, Lyashevska O. Predictive analytics with gradient boosting in clinical medicine. Ann Transl Med. 2019;7(7):152. doi: 10.21037/atm.2019.03.29 31157273 PMC6511546

[31] Aliya A, Yurf Asghar S, Danish Khan Yousafzai A, Haider Bangash A, Mohsin R, Fatima A, et al. Prediction of In-Hospital Mortality Among Heart Failure Patients: An Automated Machine Learning Analysis of Mimic-III Database. Am Heart J. 2022;254:261. doi: 10.1016/j.ahj.2022.10.069

[32] Fadavi N, Fadavi N. Early recognition of Parkinson’s Disease through acoustic analysis and machine learning. arXiv preprint arXiv:2407.16091. 2024.

[33] Safaei N, Safaei B, Seyedekrami S, Talafidaryani M, Masoud A, Wang S, et al. E-CatBoost: An efficient machine learning framework for predicting ICU mortality using the eICU Collaborative Research Database. PLoS One. 2022;17(5):e0262895. doi: 10.1371/journal.pone.0262895 35511882 PMC9070907

[34] Demsar J. Statistical Comparisons of Classifiers over Multiple Data Sets. J Mach Learn Res. 2006;7:1–30.

[35] Dharmarathne G, Bogahawaththa M, McAfee M, Rathnayake U, Meddage DP. On the diagnosis of chronic kidney disease using a machine learning-based interface with explainable artificial intelligence. Intelligent Systems with Applications. 2024;22:200397. doi: 10.1016/j.iswa.2024.200397

[36] Dharmarathne G, Jayasinghe TN, Bogahawaththa M, Meddage DPP, Rathnayake U. A novel machine learning approach for diagnosing diabetes with a self-explainable interface. Healthcare Analytics. 2024;5:100301. doi: 10.1016/j.health.2024.100301

[37] Nohara Y, Matsumoto K, Soejima H, Nakashima N. Explanation of machine learning models using Shapley additive explanation and application for real data in hospital. Comput Methods Programs Biomed. 2022;214:106584. doi: 10.1016/j.cmpb.2021.106584 34942412

[38] Kim JH, Kwon YS, Baek MS. Machine learning models to predict 30-day mortality in mechanically ventilated patients. J Clin Med. 2021;10(10):2172. doi: 10.3390/jcm10102172 34069799 PMC8157228

[39] Mamandipoor B, Frutos-Vivar F, Peñuelas O, Rezar R, Raymondos K, Muriel A, et al. Machine learning predicts mortality based on analysis of ventilation parameters of critically ill patients: multi-centre validation. BMC Med Inform Decis Mak. 2021;21(1):152. doi: 10.1186/s12911-021-01506-w 33962603 PMC8102841

[40] Prybylo M, Haghighi S, Peddinti ST, Ghanavati S. Evaluating privacy perceptions, experience, and behavior of software development teams. arXiv preprint arXiv:2404.01283. 2024.

[41] Santos S, Breaux T, Norton T, Haghighi S, Ghanavati S. Requirements satisfiability with in-context learning. arXiv preprint arXiv:2404.12576. 2024.

