# Supplementary material for: A machine learning-based prediction of hospital mortality in mechanically ventilated ICU patients
Source: PLoS One. 2024 Sep 4;19(9):e0309383. doi: 10.1371/journal.pone.0309383 (PMC11373795; doi:10.1371/journal.pone.0309383)
Supplement: S1 Graphical abstract — (PDF) [file pone.0309383.s001.pdf]

A Machine Learning-Based Prediction of Hospital Mortality in Mechanically Ventilated ICU Patients

BACKGROUND

ICU Patients on Mechanical Ventilation

High Mortality Risk

Increased Healthcare Costs

Critical Need for Accurate Predictions

METHODOLOGY

Data Preprocessing and Modeling

MIMIC-III Database

25,202 patients

Feature selection: Backward Elimination, Lasso Method
Preprocessing: Mean Imputation, SMOTE for Class Imbalance
Models: CatBoost, XGBoost, Decision Tree, Random Forest, SVM, KNN, Logistic Regression

OUTCOME

Model Performance and Key Findings

Cohort Comparison

AUROC, Accuracy, Precision, Recall, F1-Score

Calibration Plots

SHAP Analysis

The most significant feature identified was age. Our best model, CatBoost, achieved an AUROC of 0.862, exceeding the literature's best AUROC of 0.821. This highlights our model's enhanced accuracy in predicting hospital mortality in mechanically ventilated ICU patients.
