# Supplementary material for: A machine learning-based prediction of hospital mortality in mechanically ventilated ICU patients
Source: PLoS One. 2024 Sep 4;19(9):e0309383. doi: 10.1371/journal.pone.0309383 (PMC11373795; doi:10.1371/journal.pone.0309383)
Supplement: Attachment — Submitted filename: Response to Reviewers.pdf [file pone.0309383.s003.pdf]

07/25/24

Dear Editor,

Thank you for giving us the opportunity to submit a revised draft of our manuscript titled “A Machine Learning-Based Prediction of Hospital Mortality in Mechanically Ventilated ICU Patients” to *PLOS ONE.* We appreciate the time and effort that you and the reviewers have dedicated to providing valuable feedback on our manuscript. We have been able to incorporate changes to reflect all the suggestions provided by the reviewers. We have highlighted the changes within the manuscript. Both the tracked version and the clean version of the revised manuscript have been supplied with our submission. The following is a point-by-point response to the reviewers’ comments and concerns.

Sincerely,
Maryam Pishgar

**Reviewer 1 Comments**	**Response to Reviewer**
1. This paper does not include a graphical abstract. It is recommended to include a graphical abstract.	Thank you for your valuable suggestion. We have now included a graphical abstract to enhance the presentation and understanding of our paper. This has been added to our submission.
2. What approach would you suggest comparing the mechanically ventilated ICU patients of Chronic heart failure with patients with liver failure?	Thank you for your insightful comment. We included certain disease counts in our model not for comparison but to enhance predictive accuracy. This decision was guided by existing literature that highlights the relevance of these diseases, and by expert advice on their strong association with mortality in mechanically ventilated patients. Including the number of these diseases is expected to significantly improve the model's performance. We have made this point clear in our paper by adding a detailed explanation in the Methodology section.
3. How could you improve the accuracy of your predictive model? What are the limitations?	Thank you for your insightful comment. We have added a section to the manuscript summarizing potential strategies to enhance the accuracy of our CatBoost predictive model. These strategies include using grid search or Bayesian optimization for optimal

	parameter identification, feature engineering based on domain knowledge, and incorporating ensemble methods to combine predictions from multiple models. While these approaches can significantly boost accuracy, they also introduce certain limitations such as increased computational complexity and potential bias. We have detailed these strategies and their implications in the manuscript.
4. What outcomes would you expect in your validation studies?	Thank you for your insightful comments. We added the need for future validation studies using external datasets in the manuscript, specifically in the limitations section. We emphasize the importance of testing our model's performance metrics, such as AUROC, accuracy, and F1-score, on new data to confirm consistency and identify any biases or limitations across different patient populations. Additionally, we highlight the integration of real-time data streams and continuous learning models to ensure the model remains current with the latest clinical data and practices, enhancing its predictive accuracy and clinical utility.
5. Compare your model with similar models in literature and elaborate more in the discussion section.	Thank you for your valuable feedback. In response, we have revised the discussion section to include comparisons with four additional relevant studies. These studies employ similar models and approaches for predicting ICU patient mortality. We have specifically mentioned the AUC and the number of features used in their models to highlight the improvements made by our approach.

**Reviewer 2 Comments**	**Response to Reviewer**
1. The literature review is not thorough, needs to improve a lot; there should be a flow of ideas clearly showing the gap	Thank you for your valuable feedback. We have added discussions on the high mortality rate associated with mechanical ventilation (MV) and the necessity of studying this relationship to develop predictive models that aid in early intervention. We also highlighted the limitations of current studies, which often do not fully utilize advanced machine learning algorithms or address class imbalance issues effectively. We then outlined how our study addresses these gaps by implementing a thorough feature selection process and effective methods for managing class imbalances, ultimately providing a more accurate and practical tool for predicting MV patient mortality.
2. Why did the authors select these models was not clear to me	Thank you for your valuable comment. We have clarified the rationale for selecting these models in the manuscript. We chose six widely used machine learning algorithms based on their frequent selection in the literature to provide a comprehensive comparison and evaluate the effectiveness of our methods. Additionally, we included CatBoost for its advantages in handling categorical features and reducing overfitting. More explanation has been added to the paper to address your concern.
3. Improve the clarity of the figures, present versions are default generated ones from python	Thank you for your valuable feedback. We have improved the clarity of the figures by customizing them. The updated figures are now clearer.
4. Rather than suddenly jumping to SHAP, it is good to mention the applicability of SHAP in medical domain, for you references:	Thank you for your valuable feedback. In response to your comment regarding the sudden introduction of SHAP, we have revised the manuscript to include a discussion on the applicability of SHAP in the medical

https://www.sciencedirect.com/science/article/pii/S2667305324000723<br><br>https://www.sciencedirect.com/science/article/pii/S2772442524000030	domain. Specifically, we referenced two studies that utilized SHAP to explain machine learning models in medical applications, enhancing transparency and aiding clinical decision-making.
5. Future research should explore the integration of deep learning approaches, such as 269Convolutional Neural Networks (CNNs) and Recurrent Neural Networks (RNNs), " these are mostly used for time series analysis. Are they applicable for type of tabular records you used?	Thank you for your insightful comment. In this paper, we collected laboratory data using maximum, average, and minimum values over a specified period as indicators of patient conditions. However, this approach overlooks crucial time series information that can be extracted from the MIMIC-III database. We posit that incorporating time series analysis could significantly enhance the model's performance and generalizability. Time series methods can capture important temporal patterns, such as autocorrelation and trends, which are often critical in medical contexts. For instance, rapid changes in certain parameters over time are frequently associated with high mortality rates. By leveraging time series techniques to identify and quantify these temporal patterns, the model can achieve more precise and reliable predictions, potentially uncovering complex relationships between parameter dynamics and patient outcomes that static measures may miss. We have included a discussion on this potential improvement in the limitations and future work section of the manuscript.
6. Conclusion needs a revision, the present one is not good. Please summaries the main findings. Don't include limitations as you do have a separate section for that	Thank you for your valuable feedback. In response to your comment regarding the conclusion, we have revised it to more clearly summarize the main findings of our study, ensuring a concise and focused presentation of results.<br><br>Our revised conclusion now emphasizes the significant improvements in predictive accuracy and robustness achieved through advanced machine-learning techniques. It

	highlights the effectiveness of our feature selection process and the handling of class imbalances, as well as the strong performance metrics of our models.
