# Supplementary material for: A machine learning-based prediction of hospital mortality in mechanically ventilated ICU patients
Source: PLoS One. 2024 Sep 4;19(9):e0309383. doi: 10.1371/journal.pone.0309383 (PMC11373795; doi:10.1371/journal.pone.0309383)
Supplement: Attachment — Submitted filename: PONE-D-24-28799 - Review.docx [file pone.0309383.s002.docx]

**PONE-D-24-28799 – Review Report**

This paper discusses an interesting approach to machine learning-based prediction for hospital mortality in mechanically ventilated ICU patients. This analysis is crucial in ICU facility management. Hence, I recommend this paper for minor revision, and the following are a few comments to improve the paper,

- This paper does not include a graphical abstract. It is recommended to include a graphical abstract.
- What approach would you suggest comparing the mechanically ventilated ICU patients of Chronic heart failure with patients with liver failure?
- How could you improve the accuracy of your predictive model? What are the limitations?
- What outcomes would you expect in your validation studies?
- Compare your model with similar models in literature and elaborate more in the discussion section.
